# Fight, flight or finished: forced fitness behaviours in *Game of Thrones*

**DOI:** 10.1136/bjsports-2017-098170

**Published:** 2017-09-13

**Authors:** Ryan E Rhodes, E Paul Zehr

**Affiliations:** 1 Department of Exercise Science, Physical and Health Education, University of Victoria, Victoria, British Columbia, Canada; 2 Behavioural Medicine Laboratory, University of Victoria, Victoria, British Columbia, Canada; 3 Rehabilitation Neuroscience Laboratory, University of Victoria, Victoria, British Columbia, Canada; 4 Division of Medical Sciences, University of Victoria, Victoria, British Columbia, Canada; 5 Island Medical Program, University of Victoria, Victoria, British Columbia, Canada; 6 Centre for Biomedical Research, British Columbia, University of Victoria, Victoria, British Columbia, Canada; 7 Division of Neurology, Department of Medicine, Faculty of Medicine, University of British Columbia, Victoria, British Columbia, Canada; 8 Department of Human Discovery Science, International Collaboration on Repair Discoveries (ICORD), Vancouver, British Columbia, Canada

**Keywords:** behaviour, biology, survival

‘If you know others and know yourself, you will not be imperilled in a hundred battles; if you do not know others but know yourself, you win one and lose one; if you do not know others and do not know yourself, you will be imperilled in every single battle’

―From ‘The Art of War’ by Sun Tzu (544–496 BC)^1^

‘Storms come and go, the big fish eat the little fish, and I keep on paddling.’

―Varys ‘the Spider’ in the second season ‘*Game of Thrones*’ episode ‘The Night Lands’ that premiered on 8 April 2012.

Blood thundering in his ears, Jon Snow raises his broadsword Longclaw in a smooth arc of Valyrian steel that abruptly stops over his head only to then reverse course in a deadly slash downward onto the head of the Other and cleaving it in two. All his senses blazing with clarity, the bastard son of the late Eddard Stark, now himself leader of Castle Black, turns to face his next adversary full of power and purpose and thrumming with the epinephrine rush of combat.

Spoiler alert! This paper deals with plot points found in the GRR Martin books in the ‘Song of Ice and Fire’ series and the ‘*Game of Thrones*’ television production seasons 1–6. If you haven’t yet caught up (and why haven’t you?), please proceed at your own risk. You have been warned!

That thundering of blood, racing heart rate, sweating and sense of power are the hallmarks of the fight or flight response—the ability our nervous and hormonal systems have to energise us—briefly—for feats of courage, strength and power in the name of self-preservation. Call it the ‘epinephrine rush’, the ‘thrill of the chase’, it is all part of the acute stress response we have when faced with scenarios and situations that demand our complete focus and attention. And there is no shortage of such scenarios in *Game of Thrones*.

Both the response to fighting and the training for learning to fight rely heavily on the ‘fight or flight’ response. This activation of the sympathetic nervous system is the ability you have to respond to exercise stresses, which is a function of the level of regulating hormones in your body. Your state of balance—your physiological homeostasis—revolves around this.

Circulating hormones like testosterone, cortisol, growth hormone, insulin-like growth factors and catecholamines are very important for responses in muscle to exercise and injury. They are a crucial part of the stress response experienced by pretty much every character inhabiting GRR Martin’s fantasy world. Even the word ‘stress’—with its Latin root ‘stringere’ meaning to draw tight, strain, exert or tax—seems to come right out of an episode of ‘*Game of Thrones*’.

## Awash in a sea of hormones…

**Figure 1:**
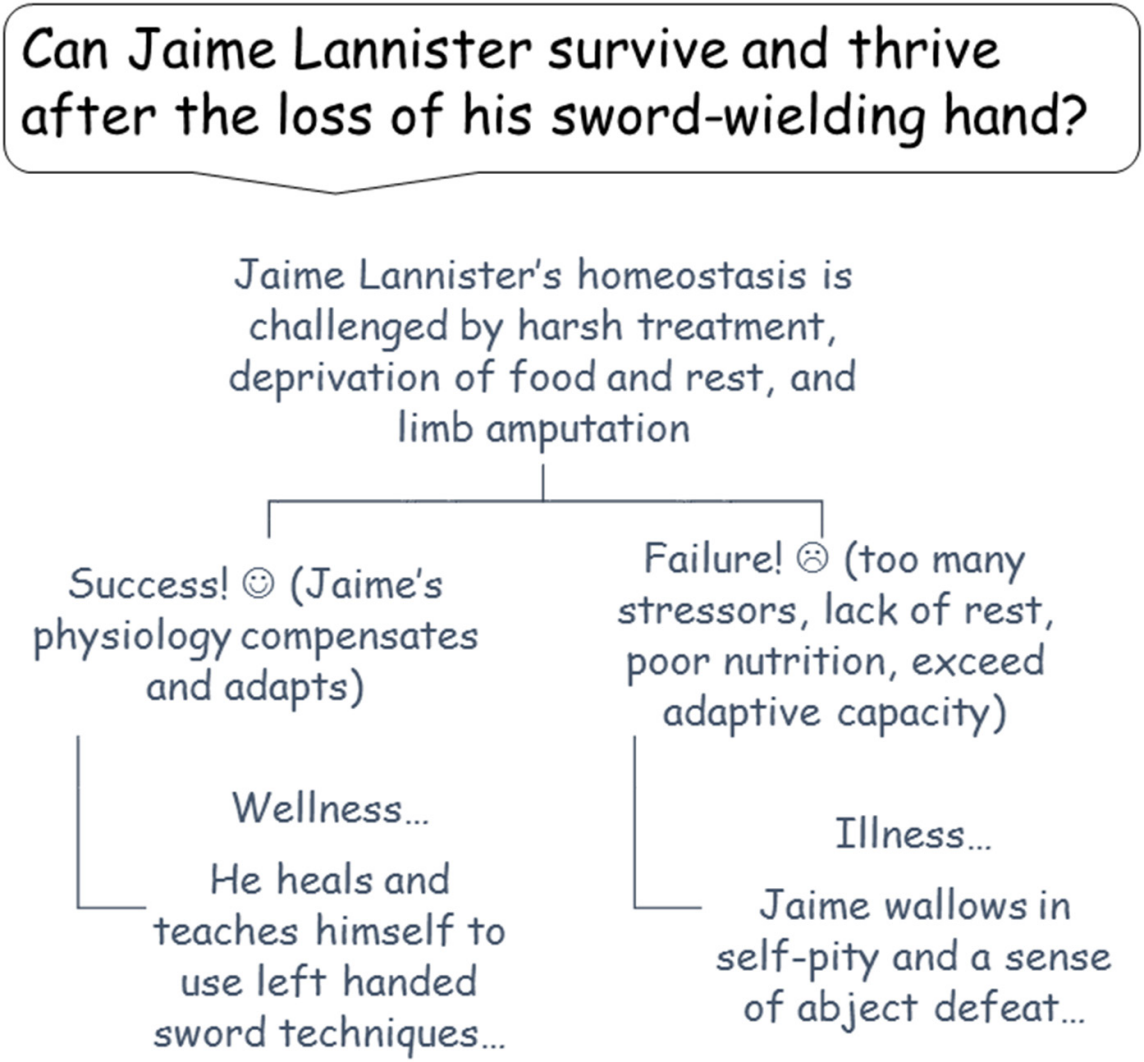
Regulating bodily balance at King’s Landing.

Thriving and surviving in our world and Martin’s hinge on the concept of homeostasis in our physiological systems. The concept of homeostasis relates to adaptive responses to change that all serve to drive the body back to its comfortable operating range (see figure 1). These include responses to extreme cold, haemorrhaging blood loss, traumatic pain and emotional distress—basically daily life in pretty much any scene in *Game of Thrones* from just about every season.

The stress response concept is part of something pioneering physiologist Hans Selye called the ‘general adaptation syndrome’ and which has three stages. The first is an ‘alarm reaction’ which mimics the ‘fight or flight’ response. The second stage is one where adaptation occurs, and there is an adjustment and resistance to the stressor so that the effect of the stressor is reduced. If adaptation is insufficient or if the stressor is too large, we enter the third stage which is one of exhaustion and cell death.

A lot of how the bodies of the characters in Martins’ *Game of Thrones* respond to daily exercise stress, stress response and various hormones. Tiny formations at the base of your brain called the pituitary and the pineal gland are important in all this. So is testosterone and is likely one of the hormones that you think of immediately.

Testosterone is a very important mediator for how the body responds and adapts to exercise stress, and levels of circulating testosterone are elevated during and after exercise. As with growth hormone, the effects are largest with more strenuous and prolonged exercise. Central in all of this are the catecholamines—those substances like epinephrine—that give us such a rush and the power to perform in fight or flight. They also have the power to make us want more and more of them in our bodies.

## Fight, flight or finished as it applies to some ‘*Game of Thrones*’ characters

While the fight or flight response describes the energy systems provided by our nervous and hormone systems, we can extend this to an analogy of survival conduct in Westeros. The fight or flight system guides behaviour in ways that either spell continued existence or certain death in *Game of Thrones*. George R R Martin’s world is a brutal survival of the fittest landscape, where characters have to possess keen senses or they are dispatched to their deaths. Those characters who adapt can thrive, but those who fail to adapt are finished.

**Figure 2:**
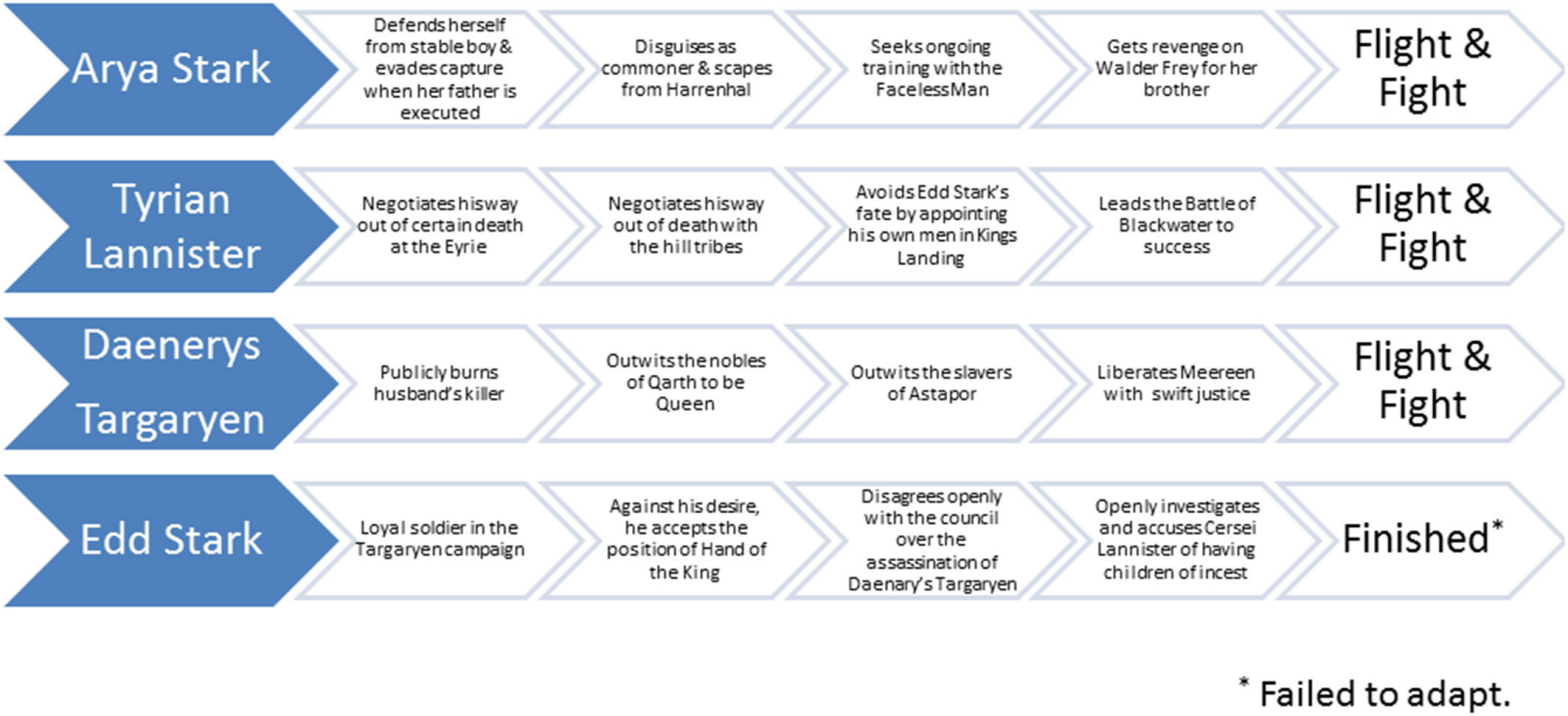
Some characters who show adaptability to fight (or not so much) to the finish.

Of course, like any theatre of war and politics, victory requires good luck and the support of background teammates. It also does not hurt to be on the winning side of magical forces in the Seven Kingdoms or across the seas in Essos, Qarth and Dothrak. Nevertheless, many of the very best and most decisive moments in *Game of Thrones* come down to individual character actions that follow fight, flight or finished consequences. While *Game of Thrones* features a vast number of these through its pages and on screen, below we highlight exemplars of each (see figure 1 and 2).

### Fight

Physical ability and fighting prowess are generally essential characteristics for survival in the ever warring Seven Kingdoms of Westeros and beyond. While Varys ‘The Spider’ and Petyr ‘Littlefinger’ Baelish do show that there are other ways to survive in this world with almost no physical ability, skill in combat is a massive advantage to survival. Three characters epitomise the use of fighting skills as a means of survival in combat and future intimidation, thus reducing the need to prove oneself at every possible conflict.

Jaime Lannister, eldest son of Lord Tywin, the powerful Lord of Casterly Rock and patriarch to the House Lannister, is generally considered the best swordsman and one of the bravest men in the Seven Kingdoms. Due to these skills, he became the youngest member of the Kingsguard in history. While his martial prowess has kept him alive in many battles throughout Westeros, his murder of the Mad King Aerys branded him the notorious Kingslayer. This has served to further fuel his lethal or treacherous reputation depending on how one views the act.

Gregor Clegane is the elder brother of Sandor Clegane (the Hound) and a landed knight who serves Lord Tywin Lannister on retainer. He is considered the largest man in Westeros and called ‘the Mountain’ based on his enormous bulk and height. This size is also complemented by a ferocity and savagery that has made him the most feared individual in the Seven Kingdoms. Gregor is suspected (and observed) in several murders and torture throughout the Westerlands. In combat, his strength gives him a huge advantage over almost any opponent, able to overpower his enemies with brute force rather than rely on his skill. The combination of these battle tactics and his fearsome reputation has allowed him to ‘survive’ thus far in *Game of Thrones*.

Brienne of Tarth begins the *Game of Thrones* series as a Kingsguard of Renly Baratheon after winning a tournament. She possesses similar attributes to both Gregor Clegane and Jaime Lannister in that her height and size are well above normal, and she has honed superior martial skills. While her service to various families in Westeros has changed across time, she has survived through her fighting prowess, physical stature and good fortune.

### Fight and flight

While skill in combat and battlefield bravery are often necessary to survive in Westeros, the ability to anticipate conflict and manoeuvre out of harm’s way proves to be equally important in the *Game of Thrones*. This ‘flight’ behaviour is the hallmark of survival skills for characters who know when they are outmatched in physical prowess at certain points of conflict. Many characters show intellect and cunning throughout the *Game of Thrones* story, but four popular characters have represented a strong mix of battlefield bravery and manipulation when survival in on the line.

Jon Snow is the bastard son of Eddard Stark, and his illegitimate status makes him an outsider to the Starks in Winterfell. Based on this status, he chooses to join the Night’s Watch. Yet his wealthy upbringing, combat training and education with the Starks of Winterfell place him as an outsider to those of the Night’s Watch. Cast as the outsider throughout his life, Jon Snow has had to use his martial skills and his wits to survive across various situations. When taken captive beyond the wall, Jon has even needed to switch allegiance, show humility and engage in subterfuge in order to ensure his survival when he is outmatched. Snow, however, is a strong leader who also understands the importance of conveying bravery and battle prowess to his followers in the Night’s Watch. While his mix of fight and flight qualities has not always ensured loyalty from all, he has found enough allies to survive (or at least be revived) in the story thus far.

Daenerys Targaryen is the child of King Aerys II and was smuggled to safety during the fall of the king’s reign and demise, which takes place before *Game of Thrones* (season 1). Although Daenerys begins the *Game of Thrones* story as a naive character, who is seemingly exploited by her brother, she gradually learns how to become an effective leader and conqueror as she seeks to regain her family’s kingdom. Daenerys shows a mixture of swift punishment to usurpers and merciful conquests when freeing slave-based cities. She develops a strong understanding of how to use diplomacy, sexuality or force (including an army and three dragons under her command) to obtain and secure her survival beyond and within the Seven Kingdoms. This mix of qualities has allowed her to survive in the story thus far.

Tyrion Lannister is the second son of Tywin Lannister and younger brother to Jaime and Cersei Lannister. He was born a dwarf, and his mother died during his birth, which are both sources of persecution and problems. Given the importance of physicality in the *Game of Thrones* world, his father and others of high and low birthrights frequently belittle him. Furthermore, his sister and father blame him for the death of his mother. Survival for Tyrion across the *Game of Thrones* story has been from his intelligence and wit. He has a gift for determining the motives of others and using this to his advantage as well as his understanding of both sides to any conflict. Tyrion uses these skills to manoeuvre out of numerous certain death situations (eg, death sentences, corruption as hand of the king); however, he is also a brave character who will engage in combat when this is the choice for survival and to retain a brave reputation (eg, the Battle of Blackwater, battle with the Starks).

Arya Stark is the youngest daughter of Eddard Stark. She represents one of the best examples of how survival in *Game of Thrones* is based on quick decision making of when to flee from danger and when to take a stand. Arya begins her story arc as a feisty child with no interest in diplomacy or leadership but soon finds herself on the run after the execution of her father. Using disguise, building strong allies and developing her fighting skills from the Faceless Man, Arya has become one of the strongest survivors of the Stark family.

### Finished

It would be hard to find a rival for the sheer number of lead characters regularly dispatched in the *Game of Thrones*. Some come to an end through bad luck or high magic, but several characters simply fail at fighting or fleeing from various conflicts—they fail at survival. While there are lots to choose from, two cases are particularly strong exemplars of the failure to engage in the correct flight or fight behaviours.

Eddard ‘Ned’ Stark was the Lord of Winterfell, and his noble line stretched back 8000 years in the North of Westeros. He had proven himself a very capable warrior and soldier during the Targaryen campaign and thus a friend of the eventual new King (Robert Baratheon) of Westeros. While his battle prowess and honour kept him alive during that campaign, his rigid sense of the boundaries between right and wrong prove disastrous in King’s Landing as when he assumes the role of the Hand of the King. Stark is deceived and subsequently outplayed by almost everyone at the King’s court as he—alone—considers duty and justice as one and the same. He eventually finds himself outmatched in battle and far behind in his understanding of the political forces in King’s Landing, which ultimately results in his death.

Joffrey Baratheon claimed the throne from his legal father, Robert, but he was actually the illegitimate son of Jaime and Cersei Lannister. Joffrey repeatedly demonstrated poor fight and flight behaviours before and throughout his troubled reign. Ultimately, he survives in *Game of Thrones* as long as he does simply due to his noble heritage and the assortment of vicious and clever characters who seek to retain him on the throne for their own ends. In terms of his lack of fighting bravery, Joffrey is bested by Arya Stark early in the story during a swordplay scuffle and proves no match for leading the Battle of Blackwater. He showed little courage during upheavals in King’s Landing among the crowds and frequently relied on his Kingsguard to remove him from danger. King Joffrey also shows no sense of when to avoid conflict and absolutely no mastery of subterfuge or deception. His taunts of his uncle Tyrion, the torture of Sansa Stark and other members/guests of the court, as well as the belittling of many common folk were all made in the open. Although Joffrey succumbs to a poison that could have dispatched any monarch, his behaviours ensured that almost anyone in King’s Landing had motive to remove Joffrey from the throne.

## **Does watching and reading *Game of Thrones* activate our own fight or flight systems?** Mirror, mirror in the brain—can seeing shape thinking?

Back in the early 90s, di Pellegrino and colleagues at the University of Parma, Italy, made a chance observation that revolutionised neuroscience.^2^ It had been known for a long time that neurons (premotor cortex) in the part of the brain that sends inputs to the final output centre for movement (motor cortex) were active to tune movements. While recording from this part of the brain in macaque monkeys, the Italian researchers discovered that those same neurons in the premotor cortex active during a monkey’s own movement were also active during observation of another monkey doing the same action!

Thus, the concept of the ‘mirror neuron’ system was born, a name that came from the way that a visually observed movement seemed to be reflected in the brain representation of the same movement in the one doing the observing. Since this discovery, extensive and convincing evidence of this ‘mirror neuron’ system and related neurons has accumulated in many non-human animals. In humans, the evidence is less direct but very compelling that we also have elements of mirror neuron activity in our brains.^3^

Here, the relevance of the mirror neuron system is that they are activated by actions regardless of whether you are doing something, anticipating doing something or watching someone else do something. Mirror neurons basically help enable us to predict the consequences of our own actions. This raises the possibility that this system might contribute to our (in)ability to understand the meaning of the actions of others.

Since understanding what others do is central to all our social interactions—and paramount in *Game of Thrones*—this has implications for understanding and communication between individuals. Perhaps this system is extremely highly functional in those who are successfully thriving in *Game of Thrones* (such as Tyrion Lancaster) and very poorly functional in those who were so ineptly dysfunctional (such as Joffrey Baratheon)?

Clearly, George R R Martin’s rich detail, interesting landscapes and dynamic yet tragic characters move viewers and readers on many levels. One possible mechanism for the appeal is the vicarious experiences *Game of Thrones* provides. Albert Bandura, one of the most influential psychologists of the 20th century, demonstrated how people can gain much from observing others.^4 5^ While personal mastery or failure experiences are considered the single most powerful influence on our thoughts and future actions, vicarious experience through watching the actions of others—particularly those we can make a connection with—is thought to be the next most powerful form of influence. Thus, if we identify with Jon Snow’s aggression as he clashes with wildlings or Tyrion Lannister’s shame as he is being insulted by his father, these experiences may be very influential on our own thoughts, feelings and actions.

Bandura’s^6^ theory is typically not examined within the context of popular television or literature. Indeed, much of the work on the power of vicarious experience is in health behaviour intervention (eg, exercise classes with similar others), psychotherapy (eg, watching a person similar to you handle a spider) or education (eg, classrooms with students of similar abilities). Still, vicarious experiences are likely to occur through popular media with great frequency, and the thrills of brutal adventure shows are likely to fulfil certain human needs. Edward Deci and Richard Ryan,^7^ for example, suggest that behaviours are performed to fulfil three basic human needs that ultimately create passionate motivation and flourishing: autonomy, competence and relatedness.

The need for autonomy refers to the desire to be self-initiating in personal behaviour. This suggests that the freedom to choose our entertainment itself is important. Despite our fascination with the subject matter, *Game of Thrones* is not for everyone! The need for competence reflects a desire to interact effectively within an environment. This is likely a critical factor in the appeal of vicarious experience in *Game of Thrones* as we see heroes vanquish villains and sit on the edge of our seats as we read whether Tyrion will escape what seems like certain death. Finally, the need for relatedness reflects the fact that individuals want to feel connected to others.^8^ *Game of Thrones*, at this juncture, represents a cultural event where fans can interact through discussion of the material.

Finally, watching or reading *Game of Thrones* may provide a kind of catharsis to activate our flight or fight systems from the safety of the couch. Catharsis comes from the Greek, meaning ‘purification’ or ‘cleansing’,^9^ and was originally applied to understanding emotional and aesthetic creation through art forms. The term has now been used in several different streams to describe the experience of deep emotion brought on via some other, rather than authentic, means.

There is definitely much debate in whether media can help abate aggression or whether it actually contributes to it.^10 11^ One thing is for certain, however; the vicarious experience of activating your flight or fight systems from the couch will not have the same behavioural health outcomes as activating this system in real life. The consequences of sustained sedentary behaviour has been described as ‘the new smoking’ in terms of its overall poor health side effects.^12 13^ All-cause mortality, cardiovascular disease and cancer mortality are 1.2 times more likely among those who engage in prolonged sedentary behaviour.^14^ The effects also get a lot larger for those who do not engage in any moderate to vigorous physical activity as this is associated with over 25 chronic conditions and poor mental health outcomes.^15^ Overall, this suggests that while *Game of Thrones* may fulfil our basic needs for fight or flight from our living room, we would all be served well to get out and have some safe adventures of our own from time to time.

## Addicted to life in the Middle Ages?

Being alive and staying alive in Martin’s landscape means performing at a high level on a daily basis. Battles, attacks, traps and ambushes are regular occurrences. And the rush of dealing with each and every one, while very stressful, could also be very addictive. It has been known for quite some time that our human brains are ‘wired’ for many addictive behaviours that notably include drugs like cocaine, nicotine and alcohol and also activities like exercise.

In a way, we really are wired for work and exactly where in the brain comes from some work on those ubiquitous laboratory mice. Back in 2003, Justin Rhodes, a zoologist at University of Wisconsin-Madison, and his colleagues studied two strains of mice, one that had the ‘normal’ interest in running on a wheel and one that were bred to be extra motivated to run.^16 17^ He and his team observed and monitored the two groups for 6 days of exercise bouts and found that the ‘extra motivated’ group had run about three times the distance of the other group.

The interesting thing is what they did next—they blocked half of the running wheels from working so it then became impossible for half the mice in either group to exercise. They then examined activity in brain circuits that are related to ‘reward’ activity in the brain. These are the same reward circuits that are so powerfully activated by opiates like cocaine and morphine. These regions are also highly activated when rodents become addicted to opiates and then denied their ‘fix’—they crave the drug.

Basically, the mice could not get the running fix needed to activate the cascades of neurohormones in the brain that would activate the reward circuitry. They were addicted to running but blocked from doing it. How much this exactly parallels our human brain circuits remains to be fully explored.

But if you simply flip exercise with fighting to stay alive or fleeing for your life as happens in *Game of Thrones*, you make the argument that everyone in those times was actually in need of the stress fix to feel ‘normal’. Maybe this is why they constantly seek conflict and intrigue—to keep the reward circuitry fully functional!

This also may explain why we love to read the books and watch the shows so much. We get a vicarious fix of the stress and thrills that happen to the characters without the real danger. Whether we are the control mice of Justin Rhodes’ study or the exercise-addicted ones is up to our own personal history and genetics. But understanding that the fitness behaviours forced onto the characters in the *Game of Thrones* are actually still biologically part of our makeup is an important realisation. Those same forced fitness behaviours that keep our favourite characters from meeting unfortunate early ends could also be usefully channelled by us to increase our own activity time.

So, maybe the next time we rewatch an episode we might do it from the (dis)comfort of a treadmill or stationary bike. We can combine both fantasy physiology and real-life neurochemistry in a clever combination that would make even Varys ‘The Spider’ proud.
